# Serial dependencies in motor targeting as a function of target appearance

**DOI:** 10.1167/jov.24.13.6

**Published:** 2024-12-06

**Authors:** Sandra Tyralla, Eckart Zimmermann

**Affiliations:** 1 Institute for Experimental Psychology, Heinrich Heine University, Düsseldorf, Germany

**Keywords:** saccadic adaptation, serial dependency, uncertainty, gaussian blobs

## Abstract

In order to bring stimuli of interest into our central field of vision, we perform saccadic eye movements. After every saccade, the error between the predicted and actual landing position is monitored. In the laboratory, artificial post-saccadic errors are created by displacing the target during saccade execution. Previous research found that even a single post-saccadic error induces immediate amplitude changes to minimize that error. The saccadic amplitude adjustment could result from a recalibration of the saccade target representation. We asked if recalibration follows an integration scheme in which the impact magnitude of the previous post-saccadic target location depends on the certainty of the current target. We asked subjects to perform saccades to Gaussian blobs as targets, the visuospatial certainty of which we manipulated by changing its spatial constant. In separate sessions, either the pre-saccadic or post-saccadic target was uncertain. Additionally, we manipulated the contrast to further decrease certainty, changing the spatial constant mid-saccade. We found saccade-by-saccade amplitude reductions only with a currently uncertain target, a previously certain one, and a constant target contrast. We conclude that the features of the pre-saccadic target (i.e., size and contrast) determine the extent to which post-saccadic error shapes upcoming saccade amplitudes*.*

## Introduction

When we gaze around in the environment, we perform saccade eye movements. Saccades are fast displacement of the eye ball whose function it is to bring visual objects of interest onto the region of highest retinal resolution (i.e., the fovea). Because rapid reception of sensory information is of survival value, performing accurate saccades is mandatory.

Inaccuracies in saccade landing can result from three sources, as the visual localization, motor localization of the target, or execution of the saccade might be inappropriate. After the saccade is finished, the error between saccade landing and the target reveals the movement success. The sensorimotor system monitors its performance and aims to minimize the error in saccade landing (Pélisson, Alahyane, Panouillères, & Tilikete, 2010). Oculomotor plasticity can be studied in the laboratory with the paradigm of saccadic adaptation, in which a saccade target is displaced while the eye is on flight (Hopp & Fuchs, 2004; McLaughlin, 1967; Pélisson et al., 2010). Due to visual transduction latencies, the saccade cannot be corrected online. Instead, after registering the post-saccadic error, the sensorimotor system triggers a corrective saccade to reach the desired target location (Sedaghat-Nejad & Shadmehr, 2021). Every experience of a post-saccadic error is followed by an adaptive change in the amplitude of the immediately following saccade. If the same artificial post-saccadic error is repeatedly presented, the adaptive amplitude change increases gradually until it reaches an asymptotic value (Noto & Robinson, 2001; Wallman & Fuchs, 1998). Maximal saccade adaptation minimizes about three quarter of the post-saccade error (Gillen, Weiler, & Heath, 2013; Ohl, Brandt, & Kliegl, 2011). In most experiments on saccade adaptation, the target is displaced in the same direction and distance. Only a few studies have investigated adaptive amplitude changes when the direction and distance of the target jump were determined randomly in every trial (Collins, 2014; Desmurget et al., 2000; Havermann & Lappe, 2010; Srimal, Diedrichsen, Ryklin, & Curtis, 2008). These studies found consistently that adaptive amplitude changes occur on the single-saccade level. Assessing the functional role of saccade adaptation requires knowing why post-saccadic errors accrue in natural vision. A putative source of post-saccadic errors would be eye muscle damage or fatigue (Abel, Schmidt, Dell'Osso, & Daroff, 1978; Kommerell, Olivier, & Theopold, 1976; Optican, Zee, & Chu, 1985). Because such changes would alter saccade dynamics permanently and thus produce constant post-saccadic errors, accumulating saccade adaptation would provide the countermeasure. Much more probable in natural vision, however, are inaccuracies in visual or saccadic targeting.

Visual estimates of object features are constantly relying on sensory input of the recent past. Serial dependencies are attractive biases toward similar stimuli previously experienced and have been observed in actions, perception, decisions, and memory (Cicchini, Mikellidou, & Burr, 2024; Manassi & Whitney, 2024). The first studies on serial dependencies used either visual orientation (Fischer & Whitney, 2014) or numerosity (Cicchini, Anobile, & Burr, 2014) as stimuli. However, serial dependencies have been reported for almost all visual features, such as luminance (Fründ, Wichmann, & Macke, 2014), orientation (Alais, Leung, & Van der Burg, 2017; Collins, 2019; Fischer & Whitney, 2014; Fritsche & de Lange, 2019; Fritsche, Mostert, & de Lange, 2017; Murai & Whitney, 2021; Pascucci et al., 2019; Rafiei, Hansmann-Roth, Whitney, Kristjansson, & Chetverikov, 2021; Tanrikulu, Pascucci, & Kristjánsson, 2023), color (Barbosa & Compte, 2020; Bays, Catalao, & Husain, 2009; Foster, Bsales, Jaffe, & Awh, 2017; Oberauer & Lin, 2017; van den Berg, Shin, Chou, George, & Ma, 2012), and shape (Collins, 2022; Manassi et al., 2021; Manassi, Kristjánsson, & Whitney, 2019).

Manassi, Liberman, Kosovicheva, Zhang, and Whitney (2018) showed that even spatial localization is subject to biases from the recent stimulation history. Subjects were required to localize objects in space, and their estimate shifted to the direction in which objects were previously encountered. We have shown that such dependencies also exist between saccade targeting and visual space. We found that artificial post-saccadic errors in the preceding trials modify visual target localization in the current trial (Cont & Zimmermann, 2021). Participants had to perform a saccade in the previous trial (Trial*_n_*_–__1_), and the saccade target was displaced during saccade execution. In the next trial (Trial*_n_*), subjects had to fixate and localize a briefly flashed target with the mouse pointer. Visual localizations were shifted in the direction of the previous post-saccadic target. Do these interactions between post-saccadic errors and visual and motor localization imply that visual and motor space relies on a shared resource? In a follow-up study, we first induced saccade adaptation. After adaptation was established, we clamped the post-saccadic error online to the predicted endpoints of saccades, effectively annulling the error (Tyralla, Pomè, & Zimmermann, 2023). Although saccade motor adaptation remained undisturbed by the experiences of zero post-saccadic error, visual adaptation–induced mislocalization gradually declined. A shared resource of visual and motor space would have dictated that motor and visual localization changes concomitantly. However, this was not the case, suggesting that motor errors recalibrate motor and visual space separately.

In the present study, we wondered how the visuospatial certainty of the saccade target would affect adaptive amplitude changes. Souto, Gegenfurtner, and Schütz (2016) measured the effect of uncertainty by using Gaussian blobs as targets for which the spatial constant was varied. They found little correlation between target uncertainty and saccade adaptation rates. Heins, Masselink, Scherer, and Lappe (2023) have shown that saccade adaptation can even be induced without presenting a pre-saccadic target. After training participants to perform a saccade to a visible target, they asked them to perform saccades to a location at which they expected the target to appear. After saccade execution, the target appeared at a position slightly shifted inward. Over the course of trials, saccade amplitudes adapted to the post-saccadic error and became smaller. These data show that the physical presentation of the saccade target might be irrelevant for adaptation to occur as long as an internal prediction about the position of the target exists.

In the current study, we aimed to test the influence of the saccade target visibility on serial dependencies in saccade targeting. We used Gaussian blobs as targets for which the spatial constant was varied. We manipulated target visibility separately for the pre-saccadic and the post-saccadic targets such that either the pre-saccadic or the post-saccadic target had a high spatial constant. We recorded saccade landing positions as a combined measure of perceptual and motor localization. The manipulation could affect either the perceptual localization of the target or the motor error correction. If the manipulation would affect perceptual localization, serial dependencies should be stronger if the spatial constant of the pre-saccadic target is high. Because such a target is unfocused and therefore more difficult to localize, the visual system should rely on past stimulations when estimating its position. If the manipulation would affect motor targeting, serial dependencies should decrease when the post-saccadic target has a high spatial constant. In that case the post-saccadic error should be less visible, thus inducing less amplitude change of the upcoming saccade.

## Methods

### Participants

Twenty-two subjects (mean ± *SD* age, 22 ± 2.99 years; 14 women) participated in the first experiment (“constant contrast” experiment) in three different sessions. Twenty-two different subjects (mean ± *SD* age, 23.50 ± 4.28 years; 17 women) took part in the second experiment (“adjusted contrast” experiment), again in three different sessions. Participants were native German speakers, reported to have normal vision or wore lenses during the experiment, and indicated no psychiatric or neurological diseases. Participants were recruited at the Heinrich-Heine University Düsseldorf. Experimental procedures were approved by the local ethics committee of the mathematics and natural sciences faculty of the Heinrich-Heine-University Düsseldorf (approval no. ZI01-2021-01). Written informed consent was given prior to the experiments in accordance with the tenets of the Declaration of Helsinki. They either received course credits or 10 euros per hour for participation.

### Setup

The first experiment (“constant contrast”) ran on a Mac Mini (2014; Apple, Cupertino, CA), presented on a cathode-ray tube (CRT) screen (Diamond Pro 2070; 12.9 inches, 800 × 600-pixel resolution, 120-Hz refresh rate; Mitsubishi, Tokyo, Japan). MATLAB R2016b (version 7.10.0; MathWorks, Natick, MA) and Psychtoolbox routines (version 3.0.17) were used for stimulus generation. The second experiment (“adjusted contrast”) ran on a Windows 10 computer (Microsoft, Redmond, CA) presented on an Acer XB272 screen (23.6 inches, 1920 × 1080-pixel resolution, 120-Hz refresh rate; Acer, New Taipei City, Taiwan). MATLAB R2020b (version 9.9.0) and Psychtoolbox routines (version 3.0.18) were used for stimulus generation. Subjects were placed 57 cm in front of the screen in a dark room. We used a black background (0.01 cd/m^2^). Participants placed their head in a chin rest to prevent head movements. Eye movements were recorded by a desktop-mounted eye tracker (EyeLink 1000 Plus; 1000-Hz sampling rate; SR Research, Ottawa, Canada). Participants performed the task binocularly, but only the left eye was recorded. A standard nine-point calibration routine was conducted. For measuring participants’ responses, a standard keyboard and mouse were used.

### Structure of trials

We asked subjects to perform a saccade to a target. We manipulated the relative spatial uncertainty of the pre-saccadic target (T1) and the post-saccadic target (T2). Both targets consisted of a two-dimensional (2D) Gaussian blob. T1 was shown before saccade execution. During saccade execution, we displaced the target (post-saccadic target T2). By changing spatial constant of the target intrasaccadically between two values (σ = 0.3° and σ = 1.5°) we aimed to manipulate the spatial certainty of the target. The lower spatial constant (0.3°) resulted in a more focused target that was connected to a higher visuospatial certainty. The higher spatial constant (1.5°) resulted in a broader target that was connected to a lower visuospatial certainty.

We created three session types: (a) both targets were small, (b) T1 was small and T2 was large, or (c) T1 was large and T2 was small. In the offline analysis, we took into account the influence of the previously seen post-saccadic target (T2*_n_*_–__1_) on the currently visible pre-saccadic target (T1*_n_*). For the three sessions, we therefore considered three dependencies: (a) T2*_n_*_–1_ small/T1*_n_* small, (b) T2*_n_*_–1_ large/T1*_n_* small, and (c) T2*_n_*_–1_ small/T1*_n_* large. Each session resulted in 400 trials (duration of 20 minutes each). The session order was randomized across subjects.

In two separate experiments, we varied the contrast of the target (peak luminance of the stimulus divided by maximum luminance the screen can reach) to further modify the spatial certainty of the saccade target. Therefore, our values reflect the percentage of maximum stimulus contrast that the monitor could show. In the “constant contrast” experiment, the same contrast was used for both spatial constant values, thus creating a constant stimulus contrast (contrast, ∼27%; minimum luminance, 0.01 cd/m^2^; maximum luminance, 3.2 cd/m^2^) that leads to targets with higher spatial constant appearing more luminant. In the “adjusted contrast” experiment, a spatial constant of 0.3° was paired with a higher contrast (contrast, ∼27%), and a spatial constant of 1.5° was paired with a lower contrast (contrast, ∼3%; minimum luminance, 0.01 cd/m^2^; maximum luminance, 157.7 cd/m^2^) to adjust for the higher spatial constant. Targets with a higher spatial constant then appeared less luminant compared with the “constant contrast” experiment and, therefore, resulted in a more uncertain target.

### Experimental procedure

**Figure 1.**
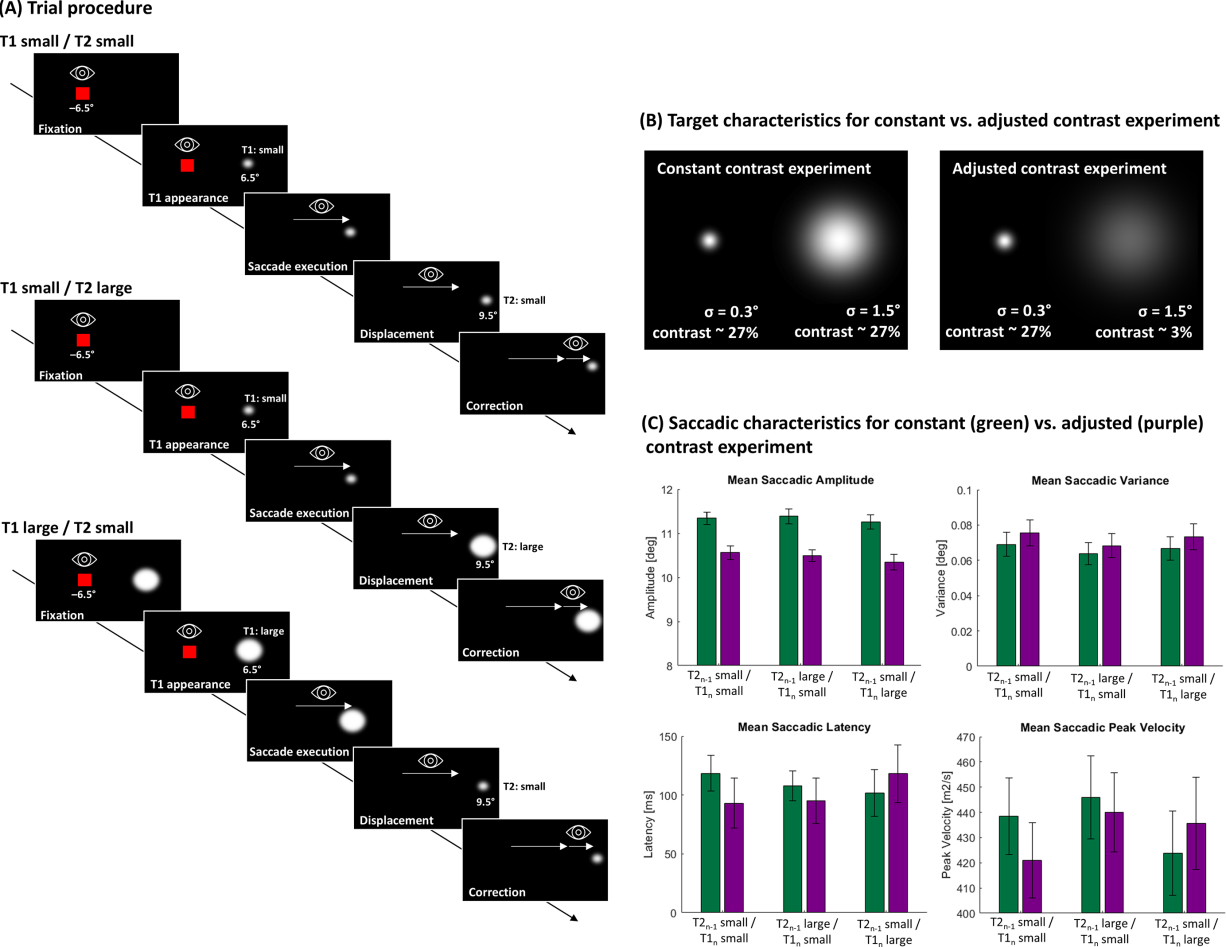
(**A**) Schematic illustration shows the procedure of one trial for each of the three sessions for the “constant contrast” experiment. Subjects performed a saccade toward the target. During saccade execution, the target was displaced in one out of six different locations (−2.5°, −1.5°, −0.5°, 0.5°, 1.5°, 2.5°). In the T1 small/T2 small session, saccadic targets were always indicated by a small diameter. In the T1 small/T2 large session, the initial target showed a small diameter and the displaced target T2 was indicated by a larger diameter. In the T1 large/T2 small session, the target identities were switched. (**B**) The stimulus characteristics for small and large targets for both experiments are specified. (**C**) The saccadic characteristics for saccadic amplitude, variance, latency, and peak velocity were specified for both, the “constant contrast” experiment (green) and the “adjusted contrast” experiment (purple). Error bars represent the standard error of the mean.

Figure 1A schematically shows the structure of a trial. Each trial began with the presentation of a red fixation square (0.55° × 0.55° diameter) on the horizontal meridian, 6.5° to the left of the screen center. The fixation square disappeared after a random duration between 500 and 1200 ms and, simultaneously, a Gaussian blob (see Figure 1B for specifications) was presented 6.5° to the right of the screen center, serving as saccade target T1. Subjects were instructed to perform a saccade toward saccade target T1 as soon as it appeared. Eye position was recorded by the eye tracker and analyzed online by the stimulus program. As soon as the eye velocity exceeded 30°/s, the target was displaced to a new saccade target position, T2. In each trial, one displacement size and direction were selected equiprobably out of six equidistant steps (−2.5°, −1.5°, −0.5°, 0.5°, 1.5°, 2.5°). The second target disappeared 1200 ms after saccade completion, and a new trial started.

### Data analyses

A trial was excluded from the analyses if no saccade was performed, the saccadic amplitude was smaller than half the required distance (i.e., 6.5°) or its peak velocity exceeded 800°/s. This resulted in a trial exclusion of ∼10% per participant. For both experiments, we computed the post-saccadic error for each trial as the difference between the actual target position of T1 (6.5°) and the saccadic amplitude. We performed linear regression analyses to examine the strength between the post-saccadic error in the previous and the current trial in each session. Student's *t*-tests against zero were conducted to investigate serial dependency effects. A one-way ANOVA with the factor session (T2*_n_*_–1_ small/T1*_n_* small, T2*_n_*_–1_ large/T1*_n_* small, T2*_n_*_–1_ small/T1*_n_* large) was calculated for both experiments separately to investigate differences in the strength of trial-by-trial influences.

## Results

**Figure 2.**
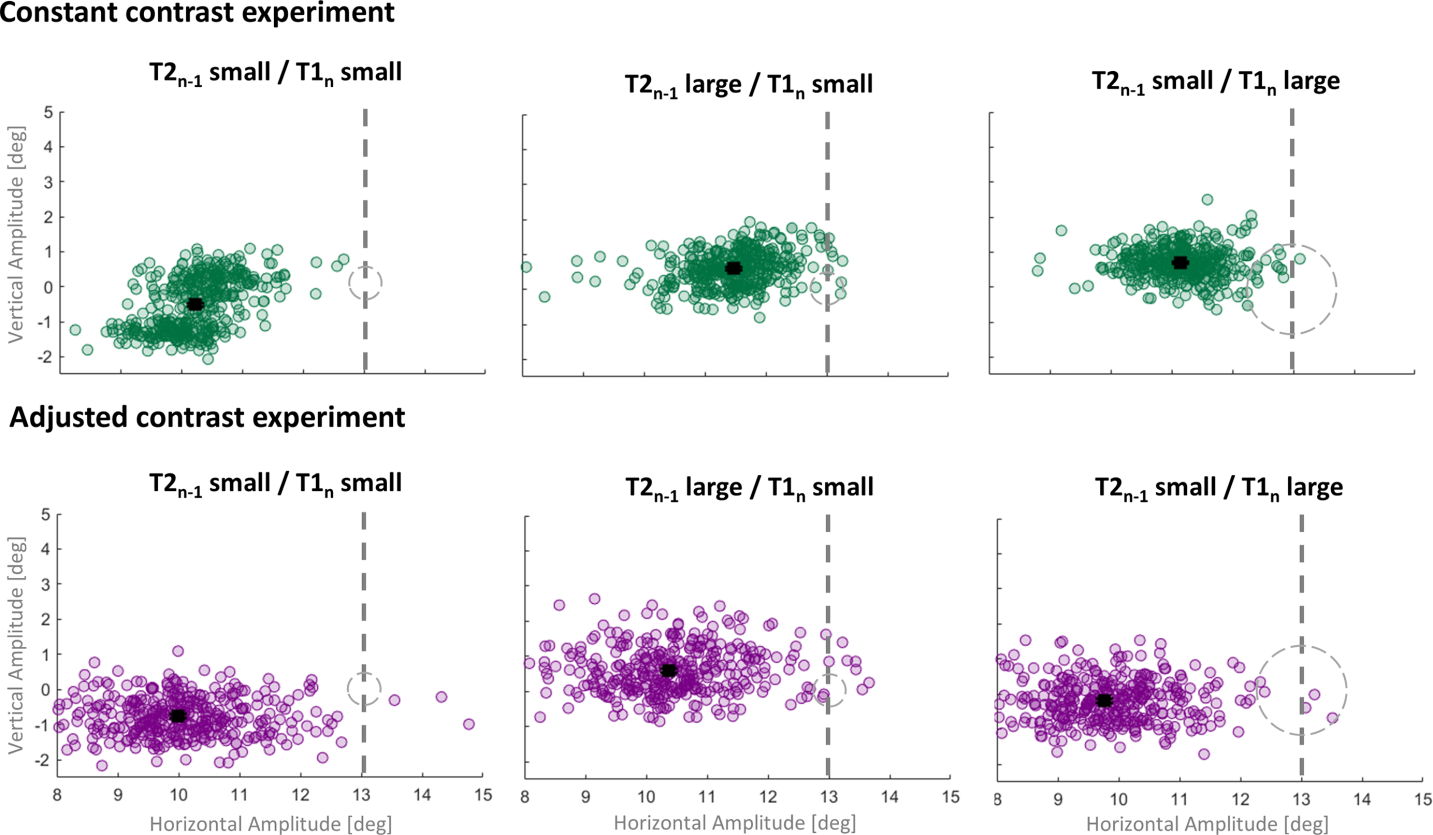
Saccadic amplitudes (degree) of two example subjects for each session and each experiment. The dashed line represents the optimal amplitude to reach the target. The empty dashed circle represent the spatial constant and position of T1. The black square represents the mean saccadic amplitude. Error bars represent standard error of the mean.

We manipulated the pre-saccadic and the post-saccadic visuospatial spatial constants of Gaussian blobs that served as saccade targets. By reducing the contrast of the larger target, we aimed to further increase visuospatial uncertainty. Figure 2 shows saccadic amplitudes for one example subject for the “constant contrast” experiment and another example subject for the “adjusted contrast” experiment for each of the three sessions. Subjects were instructed to perform a horizontal saccade of 13° (indicated by the dashed line in all panels) toward the pre-saccadic target (dashed empty circle). We varied the spatial constant of the targets, resulting in a pre-saccadic target of σ = 0.3° (followed by a post-saccadic target of σ = 0.3°; Figure 2, left panel), a pre-saccadic target of σ = 0.3° (followed by a post-saccadic target of σ = 1.5°; Figure 2, middle panel), or a pre-saccadic target of σ = 1.5° (followed by a post-saccadic target of σ = 0.3°; Figure 2, right panel). Both subjects undershot the target systematically, resulting in mean saccadic amplitudes of 10.95° for the first subject and 10.04° for the second subject, regardless of the spatial constant of the pre-saccadic target. We found a stronger saccade undershoot in the “adjusted contrast” experiment, agreeing with the results of Lisi, Solomon, and Morgan (2019) indicating that saccade undershoot magnitude scales with the visuospatial uncertainty of saccade targets.

### Post-saccadic serial dependency differences

**Figure 3.**
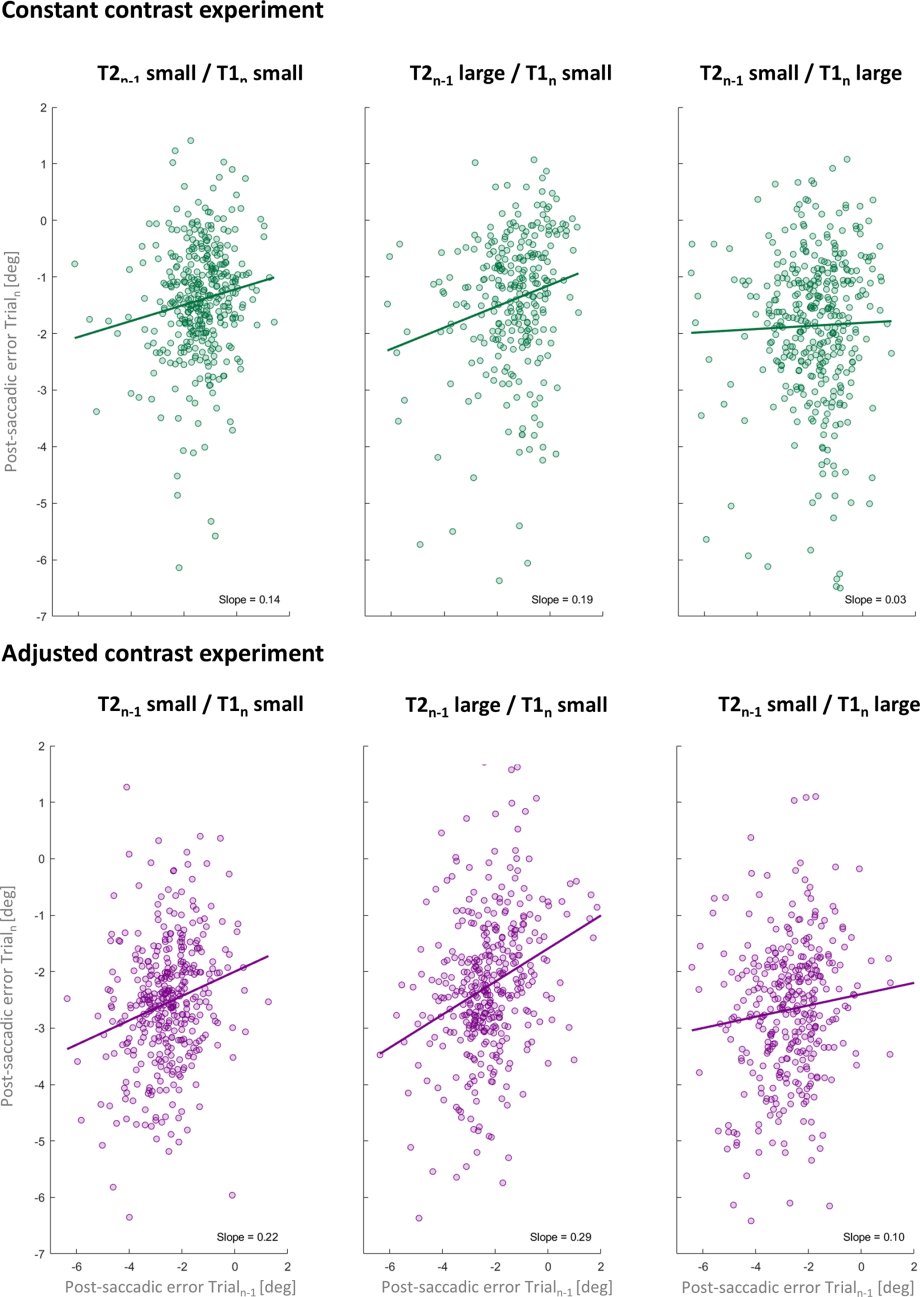
Post-saccadic error in Trial*_n_* (degree) as a function of the post-saccadic error of Trial*_n_*_–1_ of two example subjects for each session and each experiment (“constant contrast” experiment, green; “adjusted contrast” experiment, purple). Positive errors are interpreted as saccades overshooting the target, and negative numbers represent a saccadic undershoot. The positive slope (solid line) reveals that larger post-saccadic errors in the previous trial led to larger post-saccadic errors in the current trial.

We calculated the error between the actual target position and the saccadic amplitude. In Figure 3, two example subjects for each session and each experiment are presented to visualize the magnitude of saccade-by-saccade influences. Note that negative numbers indicate an undershoot of saccadic amplitude, whereas positive numbers indicate a saccadic performance overshooting the target. To investigate serial dependencies of the post-saccadic error from the previous trial (Trial*_n_*_–1_) to the current trial (Trial*_n_*), we fit a linear regression model for each subject in every session and in each experiment separately. More precisely, we took the influence of the previously seen post-saccadic target (T2*_n_*_–1_) on the currently visible pre-saccadic target (T1*_n_*) into account. Therefore, post-saccadic target T2 in the current trial (T2*_n_*) is irrelevant for the current saccadic performance: T2*_n_* is presented during saccade execution and, because of the ballistic characteristics of saccades, its amplitude cannot be changed mid-flight. We used the slopes to quantify the magnitude of saccade-by-saccade influences. Positive slopes indicate a positive serial dependency between post-saccadic errors, as larger post-saccadic errors in the previous trial led to larger post-saccadic errors in the current trial.

**Figure 4.**
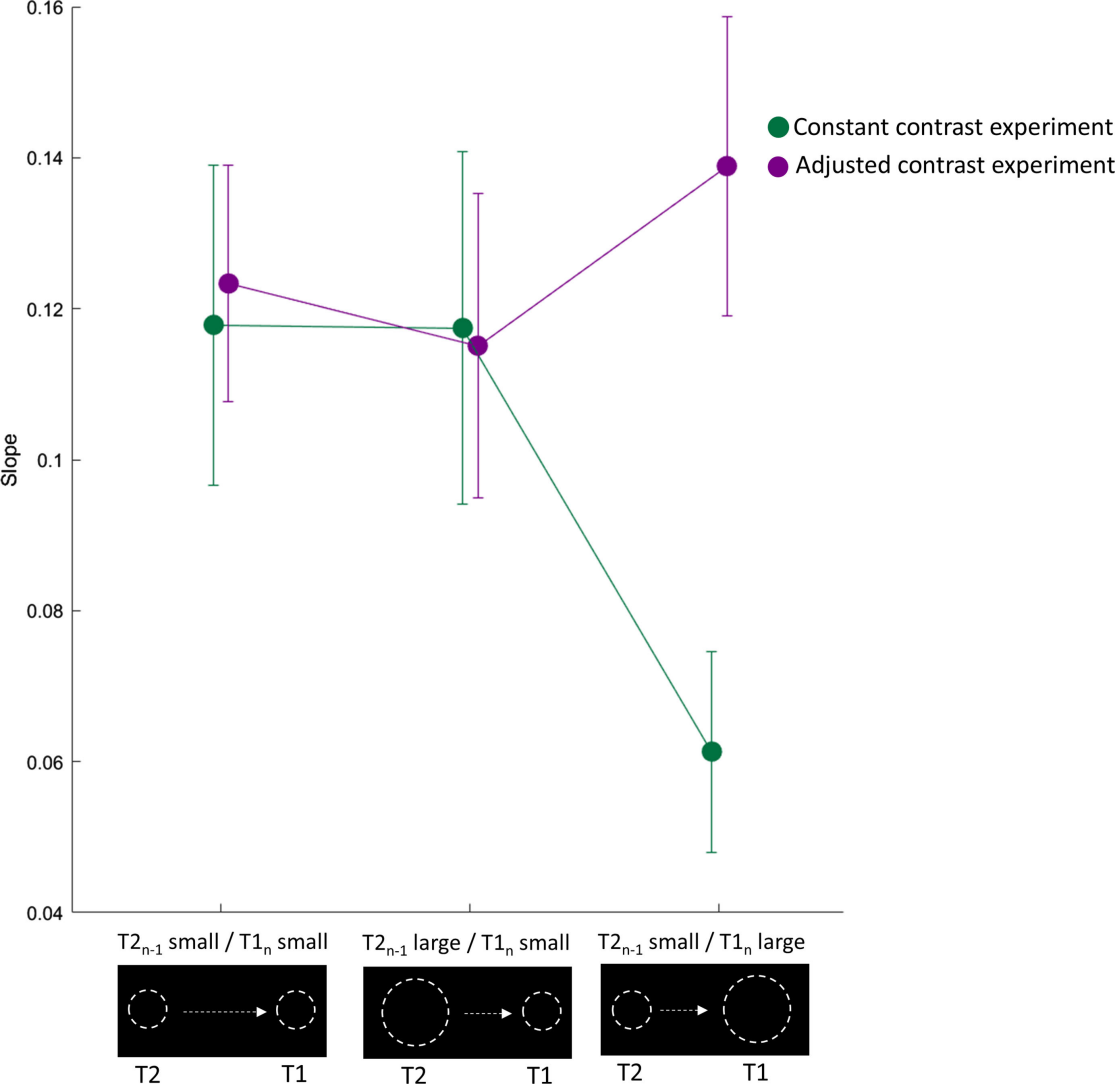
Mean slopes for the linear regression between the predictor Trial*_n_*_–1_ (either small or large post-saccadic target) and the criterion Trial*_n_* (small or large pre-saccadic target) for both experiments. Only when pre-saccadic target information was interpreted as too ambiguous did past behavior not influence current behavior, resulting in smaller serial dependency strengths. Error bars represent the standard error of the means.

Figure 4 shows the mean slopes for each session and each experiment. Descriptively, we saw a diminished serial dependency magnitude in the “adjusted contrast” experiment when the current target T1 was large and the previous post-saccadic target T2 was small. In the “constant contrast” experiment, we first investigated if influences from trial to trial could be observed independently of the session. To test this, *t*-tests against zero for the mean slopes were applied, indicating a significant serial dependency of the previous post-saccadic error on the current one, independently of the visuospatial uncertainty of the pre- or post-saccadic target (Figure 4, green; all *p* < 0.001, Bonferroni corrected). Additionally, we were interested in whether the serial dependency strength differed dependent on the uncertainty of the target. A one-way ANOVA with the factor session (T2*_n_*_–1_ small/T1*_n_* small, T2*_n_*_–1_ large/T1*_n_* small, T2*_n_*_–1_ small/T1*_n_* large) showed a significant effect, *F*(2, 42) = 4.26, *p* = 0.021. Bonferroni-corrected post hoc *t*-tests indicate that, when perceiving a highly uncertain pre-saccadic target in the current trial, preceded by a highly certain post-saccadic target, significantly less trial-by-trial influences occurred compared with the other sessions (T2*_n_*_–1_ small/T1*_n_* small: *t* = 2.54, *p* = 0.045; T2*_n_*_–1_ large/T1*_n_* small: *t* = 2.52, *p* = 0.047). No difference was found between session T2*_n_*_–1_ small/T1*_n_* small and session T2*_n_*_–1_ large/T1*_n_* small (*t* = 0.02, *p* > 0.999).

In the “adjusted contrast” experiment, we decreased target contrast with increasing spatial constant. Overall, serial dependency influences were found, independently of combination of the initial and the displaced target as *t*-tests against zero for the mean slopes indicate (Figure 4, purple; all *p* < 0.001, Bonferroni corrected). Additionally, we were interested in whether the serial dependency strength differed dependent of the visuospatial uncertainty of the target. A one-way ANOVA with the factor session (T2*_n_*_–1_ small/T1*_n_* small, T2*_n_*_–1_ large/T1*_n_* small, T2*_n_*_–1_ small/T1*_n_* large) did not show a significant effect, *F*(2, 42) = 0.54, *p* = 0.588). A pre-saccadic target with reduced spatial constant (and therefore implied lower spatial uncertainty) yielded indistinguishable serial dependency strengths compared with the other sessions.

Additionally, we performed a 2 × 3 ANOVA with the factors experiment (constant contrast, adjusted contrast) and session (T2*_n_*_–1_ small/T1*_n_* small, T2*_n_*_–1_ large/T1*_n_* small, T2*_n_*_–1_ small/T1*_n_* large) was calculated to identify differences in the serial dependence strength between the two conditions, revealing a significant interaction effect, *F*(2, 42) = 4.55, *p* = 0.016, but no significant main effect of experiment or session (all *p* > 0.265), Bonferroni-corrected post hoc *t*-tests indicated no difference among groups (all *p* > 0.156).

**Figure 5.**
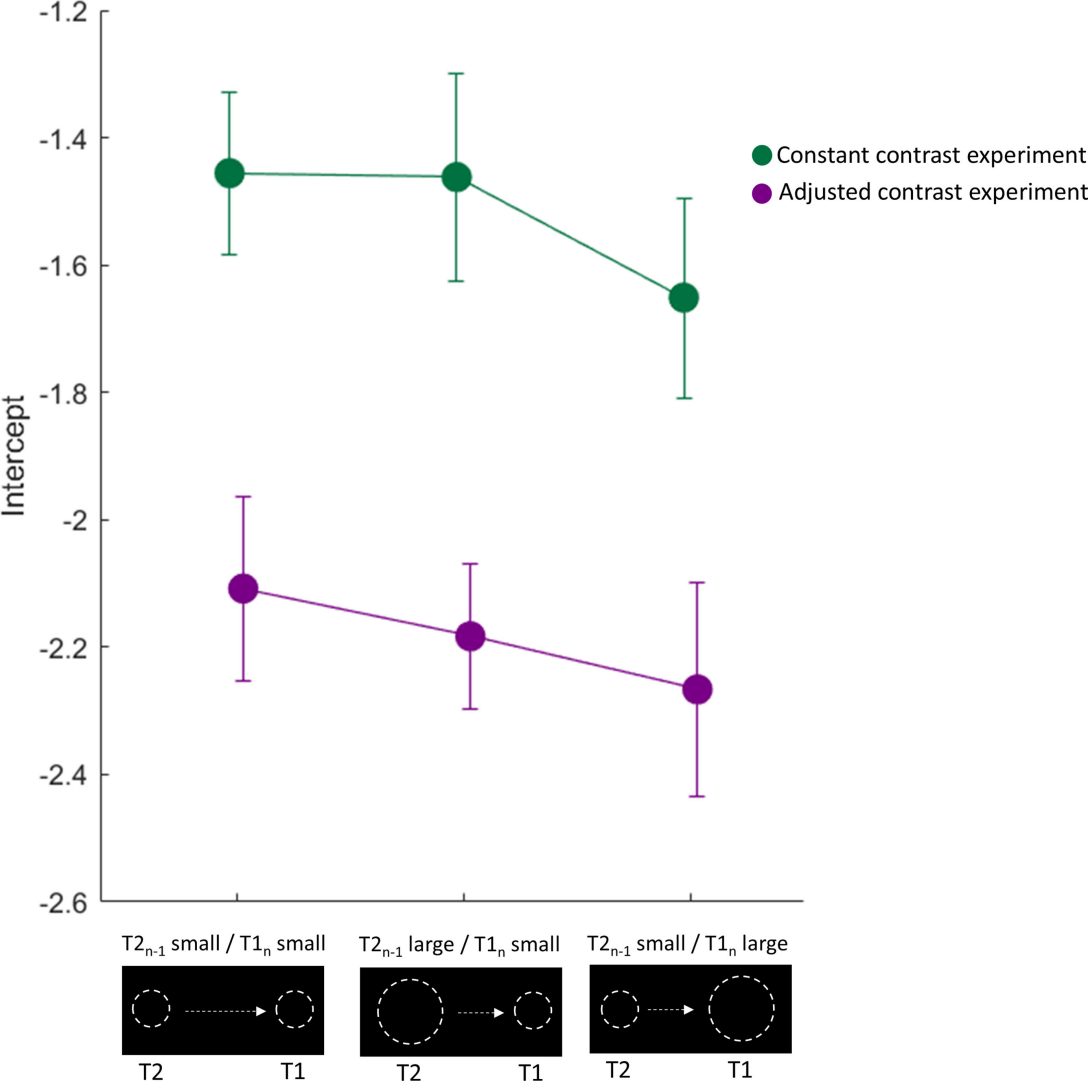
Mean intercepts for the linear regression between the predictor Trial*_n_*_–1_ (either small or large post-saccadic target) and the criterion Trial*_n_* (small or large pre-saccadic target) for both experiments. We found stronger saccadic undershoots for the “adjusted contrast experiment,” in agreement with the research of Lisi et al. (2019). Error bars represent the standard error of the means.

We performed a 2 × 3 ANOVA with the factors experiment (constant contrast, adjusted contrast) and session (T2*_n_*_–1_ small/T1*_n_* small, T2*_n_*_–1_ large/T1*_n_* small, T2*_n_*_–1_ small/T1*_n_* large) for the intercepts of the fits (see Figure 5). This analysis revealed a significant main effect of experiment, indicating a stronger undershoot for saccadic performance in the “adjusted contrast experiment” assuming no influence from the current post-saccadic error, independently of the performed session, *F*(1, 21) = 14.50, *p* = 0.003. The main effect of session and the interaction term experiment × session did not reach significance (*p* = 0.175 and *p* = 0.728, respectively).

**Figure 6.**
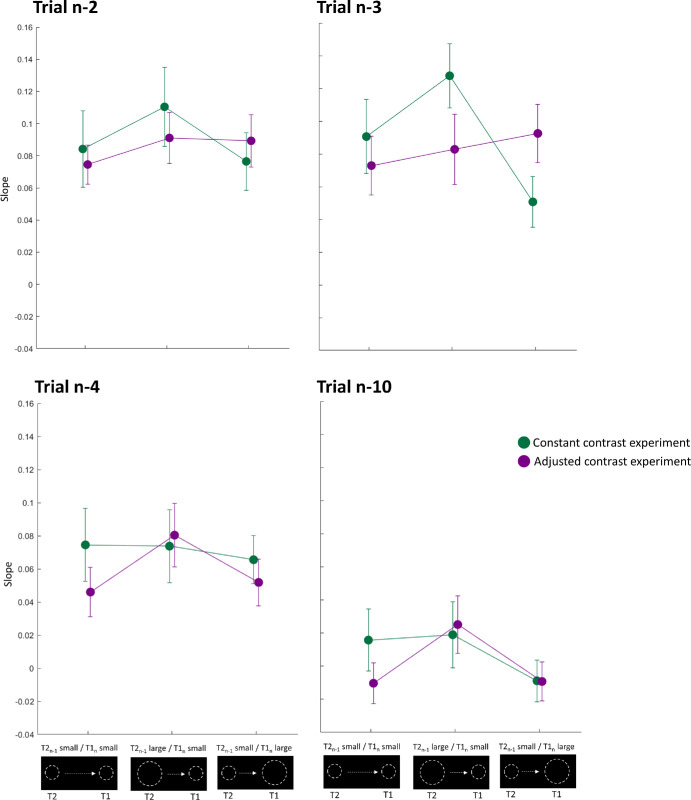
Mean slopes for the linear regression between the predictor Trial*_n_*_–__back_ (either small or large post-saccadic target) and the criterion Trial*_n_* (small or large pre-saccadic target) for both experiments. We investigated the temporal tuning of the serial dependency by investigating the influence of Trial*_n_*_–__2_ (upper left), Trial*_n_*_–__3_ (upper right), Trial*_n_*_–__4_ (lower left), and Trial*_n_*_–1__0_ (lower right). Error bars represent the standard error of the means.

We additionally analyzed the temporal tuning of the serial dependency by calculating the influence of Trial*_n_*_–__2_, Trial*_n_*_–__3_, Trial*_n_*_–__4_, and Trial*_n_*_–1__0_ (Figure 6). We performed the same 2 × 3 ANOVA with the factors experiment (constant contrast, adjusted contrast) and session (T2*_n_*_–1_ small/T1*_n_* small, T2*_n_*_–1_ large/T1*_n_* small, T2*_n_*_–1_ small/T1n large) for each *n*-back structure. Only the interaction term for the influence of Trial*_n_*_–__3_ was significant, *F*(2,42) = 4.03, *p* = 0.025; all other *p* > 0.195). Bonferroni-corrected post hoc *t*-tests indicated no difference among the groups (all *p* > 0.067).

Last, we determined if differences in the number of corrective saccades between sessions and experiments could be observed. For the “constant contrast” experiment, we identified 34.86% corrective saccades for the T2*_n_*_–1_ small/T1*_n_* small session over the whole experiment (mean amplitude, 0.49° ± 0.10°), 35.23% corrective saccades for the T2*_n_*_–1_ large/T1*_n_* small session (mean amplitude, 0.46° ± 0.10°), and 32.82% corrective saccades for the T2*_n_*_–1_ small/T1*_n_* large session (mean amplitude, 0.49° ± 0.10°). For the “adjusted contrast” experiment, the percentage of identified corrective saccades and their mean amplitudes were descriptively similar (T2*_n_*_–1_ small/T1*_n_* small, 36.80% and 0.70° ± 0.08°; T2*_n_*_–1_ large/T1*_n_* small, 35.59% and 0.58° ± 0.08°; T2*_n_*_–1_ small/T1*_n_* large, 36.85% and 0.63° ± 0.08°, respectively). A 2 × 3 ANOVA with the factors experiment (constant contrast, adjusted contrast) and uncertainty (T2*_n_*_–1_ small/T1*_n_* small, T2*_n_*_–1_ large/T1*_n_* small, T2*_n_*_–1_ small/T1*_n_* large) was calculated, revealing no significant differences among the amplitudes of the corrective saccades (all *p* > 0.203).

## Discussion

In this study, we investigated how pre- and post-saccadic saccade target uncertainty influences serial dependencies in saccade amplitudes. If a saccade target is displaced during saccade execution, amplitudes of succeeding saccades will be adaptively modified to minimize the post-saccadic error (Bahcall & Kowler, 2000; Pomè, Tyralla, & Zimmermann, 2023; Tyralla et al., 2023; Zimmermann & Lappe, 2010). We manipulated the saccade target appearance by using Gaussian blobs as targets for which the spatial constants were either small (implying a high spatial certainty) or large (implying a low spatial certainty). When both the pre-saccadic and the displaced, post-saccadic target had a small spatial constant, we observed serial dependencies with strengths comparable to those of a previous report (Cont & Zimmermann, 2021). In our main experiment, either the pre-saccadic or the post-saccadic target had a high spatial constant. We compared two experiments: If the pre-saccadic target had a high spatial constant, the post-saccadic target had a small spatial constant and vice versa. We additionally varied the contrast of the saccade.

We found that if the pre-saccadic target had a small and the post-saccadic target had a high spatial constant, serial dependencies were indistinguishable from the session in which both targets had a small spatial constant. However, if the pre-saccadic target had a high spatial constant and the post-saccadic target had a low spatial constant, the strength of serial dependencies differed drastically between the two contrast experiments. If targets had a constant contrast then serial dependencies were weak, whereas if targets had an adjusted contrast they were much stronger.

Manipulating the spatial constant of the pre-saccadic target can affect saccadic landings in several ways. On the one hand, if the pre-saccadic target has a high spatial constant, saccade landing might become more variable, thus washing out influences of the previous post-saccadic error. On the other hand, saccade landing might rely more on the previous error because the current pre-saccadic target is more difficult to localize. In that case, serial dependencies might either become relevant in the perceptual target localization or remain in the sensorimotor domain and the strength of their influence depends on the visuospatial uncertainty of the target. In both of these cases, serial dependencies should become stronger for targets with a higher spatial constant. Both of these explanations are incompatible with the observed data, as we did not find that landing was more variable for targets with a high spatial constant, nor did saccade landings take the error more into account than for a target with a small spatial constant. It is therefore unlikely that the uncertainty of the pre-saccadic target explains our data. The absence of saccade target uncertainty effects on saccade adaptation are consistent with a previous report. Souto et al. (2016) found little correlation between target uncertainty and saccade adaptation rates.

The findings lead us to conclude that it is rather the stimulus visibility and the spatial extent of pre-saccadic target T1 that determines how much the post-saccadic error (i.e., target T2) is taken into consideration. Put simply, a large saccade target allows many correct landing positions. The displaced target T2 will thus not induce adaptive changes as strong as it would have for a spatially focused saccade target T1. The post-saccadic evaluation of the landing error will be more tolerant for high contrast and large saccade targets. The tolerance built up only when pre-saccadic target T1 was large. When it was small and post-saccadic target T2 became large, no change in serial dependency strength was observed. This asymmetry demonstrates that the trans-saccadic change in the target size cannot be responsible per se for the weaker serial dependencies. One could argue that congruency between the pre- and post-saccadic target is a requirement for adaptive amplitude changes; however, we argue that a minimum target contrast is required to compare the spatial extent of pre-saccadic target T1 to the location of post-saccadic target T2. Our data revealed that the spatial extent of pre-saccadic target T1 served as an anchor in the evaluation of the post-saccadic error. Previous research established that the driving signal of saccade adaptation is the prediction error, consisting of the difference between the observed retinal and the predicted post-saccadic error (Bahcall & Kowler, 1999; Bahcall & Kowler, 2000; Collins & Wallman, 2012; Wong & Shelhamer, 2011). Heins et al. (2023) found that presenting a pre-saccadic target is not necessary to induce saccadic adaptation. Saccade amplitudes changed adaptively through the mere presence of a post-saccadic error. However, subjects could predict where the target would appear and thus also could predict the post-saccadic error. Our data show that the prediction of post-saccadic error takes into account the features of the pre-saccadic target. A large and salient target induces a spatially more distributed prediction of the saccade target than a focused target.

In conclusion, our study shows that features of a pre-saccadic target determine how strong post-saccadic errors induce adaptive amplitude changes.
